# Supplementary material for: Coordination of gaze and action during high-speed steering and obstacle avoidance
Source: PLoS One. 2024 Mar 8;19(3):e0289855. doi: 10.1371/journal.pone.0289855 (PMC10923441; doi:10.1371/journal.pone.0289855)
Supplement: Attachment — Submitted filename: Response To Reviewers.docx [file pone.0289855.s002.docx]

**Response to reviewers**

Manuscript number:	PONE-D-23-23553

Manuscript title:	Coordination of gaze and action during high-speed steering and obstacle avoidance

Authors:	Powell, Marshall, Diaz, & Fajen

We thank the reviewers for their careful reading of the manuscript and for their many helpful comments and suggestions. Our responses to each comment can be found below in blue font. Changes to the manuscript are also indicated in the marked-up version. Note that line numbers below refer to those in the marked-up version of the manuscript with changes tracked. The line numbers in the unmarked version differ because MS Word includes deleted text in its count of line numbers.

**Response to Reviewer #1 comments:**

The study explores gaze and steering behavior of drone pilots engaged in first person-view drone racing within a dense forest environment. While participants effectively completed the racing tasks, they looked less to their future waypoints than expected. Participants anticipated upcoming hoops, optimizing their steering accordingly. Overall, the paper is well-written and provides valuable insights into human navigation behavior in FPV drone racing.

My main issue revolves around statistics. I am worried about the presentation of the statistical analysis. First, the sample size in Experiment 1, involving only six subjects, and particularly the inclusion of only three subjects in Experiment 2 (one of them an author), raises concerns. PLOS One places great emphasis on appropriate sample sizes. Given the requirement for drone piloting expertise, it's understandable that recruiting a larger sample was challenging. Now that the pandemic has eased, it may be worthwhile to explore the possibility of obtaining additional subjects to enhance the robustness of the study. But I realize this might not be possible. For now, the decision to primarily focus on the results of Experiment 1 appears justified.

Second, the statistical details in the paper require further clarification. For instance, in line 316, it would be helpful to specify which post-hoc tests were employed and whether any correction method for multiple comparisons was applied. Additionally, when discussing the repeated-measures ANOVA, it should be explicitly mentioned that "condition" was treated as a between-subject factor.

Post-hoc tests were adjusted using the Bonferroni method. We added this information to the manuscript (Line 352). Condition was not a between-subjects factor, but it was inadvertently entered into the model as such in the original analysis. We thank the reviewer for catching this error and have corrected it in the revised version (see the ‘Basic Performance Measures” section of the Results, starting on Line 340). One of the tests (main effect of block on number of collisions) that was reported as statistically significant in the original version is not statistically significant (p = .07) in the corrected analysis. However, the statistical significance (or non-significance) of all other tests remains the same. As explained on Lines 328-330, these ANOVAs were run using ezANOVA rather than LME4, as in the original manuscript. The new ANOVAs also include the Greenhouse-Geisser correction where Mauchly’s test was significant.

The inverted block five really stretches the definition of repeated measures. However, it doesn't seem to be statistically different from the other blocks, except for what the authors describe as a "spurious finding." Does the analysis yield consistent results when Block 5 is excluded, apart from this "spurious" interaction?

We re-ran the analyses with Block 5 excluded. Except for the interaction in the path deviation analysis, none of the tests changed from significant to non-significant (or vice-versa). We are unsure whether the reviewer is asking us to change how we reported these analyses in the manuscript. Our preference would be to leave them as is (with Block 5 included) because we feel that separating out Block 5 would be more complicated for the reader. However, if the reviewer disagrees, we would be happy to make the change. We also became aware of a possible explanation for the elevated path deviation in the Block 5 of the Hoops Only condition and have added it to the manuscript (see Lines 416-421).

The absence of statistical tests in the "Where did subjects look?" section could be clarified. Is this related to the exclusion of subjects with imprecise eye-tracking data? For Experiment 2, the small sample size is mentioned in line 440. Were the three subjects excluded from the eye movement analysis the same three who did not participate in Experiment 2?

Yes – the decision not to report statistical tests in the “Where did subjects look?” section was due to the low number of subjects with accurate gaze data. We added a sentence to the Data Analysis section (see Lines 332-333) to explain this. Two of the three subjects in Experiment 2 were among the three from Experiment 1 with accurate gaze data (see Lines 304-305).

In the section on anticipatory steering adjustments, an LMM is used, but no specific model results are presented. It seems like the analysis involved adding the angular position of Hoop N+1 to a separate LMM and calculating an LRT. This procedure should be explicitly documented for clarity.

The reviewer’s assumptions about this analysis are correct. We added some details to better document the procedure that we followed (see Lines 641-644).

While the statistical methodology appears sound, the presentation would benefit from some refinement to ensure transparency and enable readers to have a clearer understanding of the results.

We interpreted this comment as a summary of the concerns raised in previous comments, which we addressed above. We hope that these changes clarify the procedures that we followed.

Apart from these issues, the discussion already addresses the problem of eye tracking precision, and the authors are best positioned to assess its implications for the study.

Notes:

1. In line 257, Experiment 2 is described as identical to Experiment 1, but it remains unclear to me which configuration of hoops and path from Experiment 1 was used.

We clarified that both conditions in Experiment 2 (Dense Trees and Sparse Trees) were similar to the Path Only condition from Experiment 1, but the density of trees on and near the path was manipulated (see Lines 268-273).

2. The eye tracking errors (exceeding 5 degrees for excluded subjects and averaging 3.05 degrees for the remaining subjects) are notably large. While the standards for eye tracking in VR differ from stationary eye trackers, it could be beneficial to include some discussion regarding why these errors were this large.

The Pupil Labs VR/AR extension eye tracker is mounted in a fixed position inside the HMD and is not adjustable, so it fits some subjects better than others depending on head shape. In addition, after the first couple of subjects, we implemented the calibration assessment routine (described in the “Eye Tracking” section, which starts on Line 288) that allowed us to check the average eye-tracking error and recalibrate if necessary. Lastly, we remind the reader that the mean error in the center of the display was 2.55 degrees in Experiment 1 and 2.19 degrees in Experiment 2.

3. This is a minor point, but in line 575, a comparison with car steering is drawn: While some arguments against this comparison are given, the absence of elevation in automobile steering could be acknowledged, and the possibility of testing this with a larger sample size could be pointed out. It might be helpful to clarify the intended message here?

We modified the text (see Lines 756-758) to indicate the differences in elevation control. We decided not to mention sample size here since it is acknowledged as a limitation in the very next paragraph. We also made some changes to that paragraph to clarify the intended message.

4. The paper lacks mention of motion sickness, despite the nature of the task, which could typically lead to such issues. While the Vive Pro is a solid device, motion sickness can still occur. Was motion sickness measured during the study? Were any countermeasures taken beforehand to mitigate its effects on the participants?

We added a paragraph to the methods section to address the issue of simulator sickness (see Lines 279-286). The main countermeasure taken to minimize simulator sickness was to recruit subjects with FPV drone piloting experience. This ensured that subjects were familiar with viewing simulated self-motion prior to participating in the experiment. During pilot testing, we did find that a small number of subjects with fewer hours of previous FPV drone piloting experience did encounter mild symptoms of simulator sickness within the first 5-10 minutes of the experiment. In these cases, the experiment was stopped immediately. This helped to inform the decision about how many hours of experience was necessary to participate in the experiment.

5. Figures 3 and 4 suffer from excessive JPEG compression in the PDF version.

All images meet the requirements for file type and resolution. We noticed that when Editorial Manager builds the PDF, the resolution of some of the images appears to drop. However, there is a link in the top-right corner of each image page in the PDF to the original, higher-resolution version of the image.

We thank Reviewer #1 for their many helpful comments and hope that the changes to the manuscript address their concerns.

**Response to Reviewer #2 comments:**

I read this paper with interest. The topic is timely and relevant, and the general approach is novel.

I do, however, have a number of concerns that would appear to weaken many of the conclusions of the paper.

The first set of concerns focus on the author acknowledged weaknesses. The limitations section of the paper identifies three aspects of the design that limit the study (sample size, participant expertise and error in gaze measures), however these limitations do not seem to be fully considered elsewhere. The abstract does not state the sample size, or acknowledge these weaknesses, and throughout the manuscript conclusions are drawn from the data as if these issues do not exist. In the analysis I would have been really interested to see more examination of the individual patterns of behaviour in both steering trajectories and gaze behaviours. Currently the only hint is provided in Figure 3B which shows the extent of variation in gaze performance for 2 of the 3 individuals (1 dataset is masked by the average line). Instead, the manuscript focusses on presenting group analysis for each measure which masks that this performance will be highly influenced by a single participant. Similarly the limitations section flags that eye-tracking measures had an error of 2-5deg, but this is not addressed in the eye-tracking analysis in terms of the likelihood of misclassification of target objects/regions or estimating gaze distance from path center.

We revised the abstract to include mention of the limitations. We also added text to acknowledge these limitations at several points within the manuscript (see Lines 576-578 and Lines 700-702).

We appreciate the suggestion to explore differences across subjects but are somewhat limited due to the small sample size, especially in the analyses of gaze behavior. We did, however, update the analysis of anticipatory steering behavior to include partial R^2^ curves for individual subjects (see Figure 7A and Lines 659-662). Although there were some individual differences in the degree of anticipation, especially toward the end of the segment (i.e., at 100%), the most interesting result in our opinion is that all six subjects exhibited evidence of anticipation.

It is tempting to ask if differences across subjects in the degree of anticipation are related to individual differences in measures of performance. However, with only six subjects, such an analysis is bound to be inconclusive. Indeed, we did not find any significant correlations between partial R^2^ and speed, path deviation, number of collisions, or proportion of hoops completed.

The second set of concerns tie in with the novelty of the task used and the extent to which flying a drone in the manner specified in the experiment is comparable to the experiments referenced in the introduction looking at steering along paths.

The task given to participants was: “fly as quickly as possible while staying close to the path, and attempting to pass through each hoop (when present)”. This task explicitly prioritises speed over accuracy, and would appear to reduce the importance of spatial accuracy with respect to the rendered path and the hoop.

Subjects were instructed to fly as quickly as possible while staying close to the path (in the Path Only and Hoops + Path conditions), attempting to pass through each hoop (in the Hoops Only and Hoops + Path conditions), and avoiding collisions. We did not tell them anything to suggest that spatial accuracy with respect to the path or hoops was less important, and there was nothing about the task itself that prioritized speed over accuracy or vice-versa. We added a sentence to the Methods section to clarify this point (Line 250-252).

In his comments below, the reviewer raised several questions about the evidence that subjects performed the task accurately. We address each of these comments in turn below. We also want to point out here that had subjects prioritized speed over accuracy, we would have expected average speed to be closer to 100% of maximum speed, which was ~17 m/s. Instead, average speed ranged from 12-15 m/s (see Figure 2A), which is between 75% and 90% of maximum speed.

With these instructions I would expect trajectories to only loosely align to the rendered path since the priority is taking the quickest route (that passes through the hoops). The equivalent when driving would cause drivers to take the “racing line” which leads to ‘corner cutting’ (deviation away from the center of the path). This is crucial for interpreting the presented data because research shows that such instructions can alter gaze patterns – e.g Reference [16] showed that when drivers were instructed to take the racing line, gaze fell on the inside edge of the demarcated road.

The task instructions causes two problems for the manuscript in it’s current form: (A) It is unclear that the analysis of steering and gaze behaviours are sufficient/appropriate to identify whether the above behaviours occur, or to confirm whether gaze is being directed toward the paths actually taken (rather than the paths rendered on the screen); and (B) The conclusions that gaze is only weakly coupled with steering could simply be a result of the reduced importance/relevance of spatial information in this task.

The reviewer raises an interesting point. None of the analyses that were reported in the original version of the manuscript could rule out corner-cutting behavior. Furthermore, fixating points that lie off the path while cutting the corner would not necessarily be inconsistent with the general hypothesis that people look where they want to go and steer in the direction of gaze. We made several changes to the manuscript to address this point.

First, as we explained in response to the previous comment, we clarified (see Line 250-252) that there was nothing in the instructions to indicate to subjects that they should prioritize speed over accuracy. The fact that subjects consistently flew below maximum speed indicates that they balanced speed and accuracy.

Second, we added a new analysis to show that subjects did not exhibit corner-cutting behavior. This analysis is summarized in our response to the next comment since the concern about how closely subjects followed the path was raised in more detail there.

Third, we clarified in our responses below and in the manuscript (see Lines 487-489) that the path was lined with trees and that whenever the body of the quadcopter collided with an object in the scene (including a tree), its velocity was altered to simulate bouncing off the object. As such, it was difficult or impossible on most curves to cut the corner without risking a collision.

The broader point that we’re trying to raise in this study is that in densely cluttered environments, gaze will often fall on locations other than the intended direction of travel due to occlusions. In addition, when the path is visually similar to the surrounding background (as it was in the present study), subjects have to actively search the scene to find the path, which means that some fixations will fall on locations that are off the path. This is not to dispute the idea that humans prefer to look where they want to go, but rather to question the idea that successful steering requires that they look where they want to go. The fact that subjects were able to perform the task well (maintaining a high speed while following the path and steering through hoops) suggests that successful steering is possible even when gaze is not consistently directed to the path.

I will expand in detail on some of the issues around (A) and (B):

It seems that the analysis of steering does not really show whether there is good compliance to the rendered path. The value of 0.83 m deviation from path center is reported and used to suggest that the drone remained on or close to the path, however deviation of more than +/- 0.5m from the path center could be considered leaving the path (given the whole path is only 1m wide). The fact that path deviation metrics do not differ when the path is no longer rendered (and only hoops are present) would seem to suggest that the rendered path is only weakly influencing steering because it is not prioritised in the task instructions. Further examination of the shape of the trajectories relative to the path may be able to provide a better sense of whether the drone pilots were merely taking the shortest/quickest route through each lap (as instructed), or whether trajectories are in fact constrained by the rendered path.

If subjects were heavily prioritizing speed over accuracy in path following, then we might expect them to cut the corner on sharp turns. This would be reflected in terms of an inside bias in path deviation. We ran a new analysis of signed deviation from path center, focusing on nine individual segments of the loop where the path curved to the left or right (see Figure 3A). The results are shown in Figure 3B. Positive deviations indicate an outside bias and negative deviations indicate an inside bias. In the Path Only condition, there was an inside bias on four of the nine curves (1, 3, 6, and 9) but the mean bias was less than -0.5 m only on Curve 3. Furthermore, the bias was to the outside on just as many curves (2, 4, 5, and 7). This is not surprising because the path was surrounded by trees on either side, so it was difficult or impossible on most curves to cut the corner without risking a collision. Signed path deviation was even closer to zero in the Hoops Only and Hoops + Path conditions. This makes sense because the hoops were positioned on the path, so in general, subjects could not cut the corner without missing a hoop. We added a new section to the results that includes this analysis (see Lines 471-500).

The manuscript states in a few places that “subjects performed the task extremely well” however because the priority instruction was to travel quickly it makes interpreting spatial errors difficult because there will clearly be speed-accuracy trade-offs. Trials where there were large spatial errors (>10m left/right, or 20m vertically) were halted and a new trial was run instead, though no report of how often this occurred is given making it hard to interpret whether the data are representative of most flight attempts. Number of collisions reported in Figure 2 varies, but after very high values in the first block they seem to settle to approximately 5-10 per lap - the authors interpret this as “infrequent” but I find this value hard to interpret. Compared to driving a vehicle on a road (where no collisions are tolerated) this value still seems extraordinarily high, however, it is possible that a very low threshold is being used to classify a collision (i.e. is clipping a branch/leaf on a tree a collision?) or that this could be considered low given how many potential collisions with trees/hoops there might be during a lap.

The simulation was designed such that if the quadcopter strayed too far off the path, it was automatically teleported to the start of the current lap. However, this never actually occurred in any sessions of either experiment. We added a sentence to clarify this point (Line 262). This is relevant to the reviewer’s concern because it provides additional evidence that subjects did not prioritize speed at the expense of accuracy. Again, there was nothing in the instructions to indicate to subjects that they should prioritize one over the other, and their behavior was consistent with a balance between the two criteria.

We agree that 5-10 collisions in a ~740 m stretch would be extremely high in the context of driving, but this is not a fair comparison because there is so much more clutter in the scene compared to typical roadways. Movie 1 in the Supporting Information gives the reader a clear sense of the density of clutter. In addition, any collisions, including those that involved clipping a branch or a leaf, were counted. Thus, when one considers the number of potential obstacles on or near the path and the low threshold for classifying collisions, 5-10 per lap indicates that subjects were in general successful in performing the task.

Unfortunately, I could see no definition of what constitutes a collision in the manuscript, how long a lap takes (on average), nor was it clear whether anything happened to the drone as a result of a collision (i.e. did it just fly through the collision object, or get slowed down, or stopped?). Mention is made of the drone getting stuck in branches which suggests that some collision detection was used to alter movement of the drone.

Collisions were described in the original manuscript in the Methods subsection on the virtual environment (see Lines 222-224). In the revised version, we also added a few sentences to the Design and Procedure section (Lines 253-256) to explain what happened when the drone collided with an object. It did not just fly through the object. Rather, its velocity was altered to simulate bouncing off the object. In the case of a head-on collision, the quadcopter bounced backwards and its velocity sharply dropped for a brief period. This is relevant to the reviewer’s concern because it means that there was a cost to flying too quickly and ignoring obstacles. A head-on collision could cost the subject time (at least a second) and cause them to briefly lose control of the quadcopter.

It took subjects about 60-75 s to complete a lap (see Line 150).

Related to this it is unclear whether the path/ground is itself a potential collision object – it seems likely it would need to be or the drone could pass through the ground.

Correct. If the drone collided with the ground, it was registered as a collision and the drone bounced upwards (see Line 254).

However this does then undermine a key conclusion that pilots did not need to look at collision obstacles. The fact that the ground could be considered an ‘attractor’ along 2 axes and a ‘repellor’ in the vertical axis would seem to be of potential interesting but I could not see any consideration of this characteristic (i.e. how high did the pilots fly above the ground and how consistent was this height maintained?). Depending on how collisions/spatial errors are handled, in terms of error feedback, you might expect different behaviours from the pilots – e.g. participants might be inclined to reduce spatial error/collisions if that led to slower trials, or if they knew for sure from error feedback that they had failed the task of passing through each hoop. The number of collisions approximately halved when hoops were removed (in block 5) so it seems possible that around half of collisions (~5) were with the hoops themselves, suggesting that pilots often clipped the hoop rather than passing through the centre. This also highlights an issue with the ‘hoops completed’ metric – what was the spatial cut-off for success? Did colliding with the hoop constitute ‘completion’? If there was no temporal penalty for collisions then it seem likely that trajectories will again be biased toward the quickest trajectory at the cost of passing cleanly through the hoops, which again would seem to weaken the importance of gaze and steering being tightly coupled.

The currently reported metrics would seem to reinforce the idea that the drone pilots were accepting spatial trajectory errors as a trade-off for increasing their speed completing laps. This is important since it could explain why gaze does not appear to be directed to the rendered path or hoop as much as would be expected by the literature.

As we explained in our response to an earlier comment, when the body of the drone intersected with any object in the scene (tree, branch, leaf, hoop, ground), a collision was registered and the velocity of the drone was altered to simulate bouncing off the object. This means that there was a definite penalty for colliding with objects – collisions slowed the quadcopter for a brief period and there was a temporary loss of control.

The proportion of hoops completed was based on the number of hoops through which the quadcopter passed divided by the total number of hoops, regardless of whether the quadcopter collided with the edge of a hoop before passing through. If we use the stricter definition of success that only includes hoops that were completed without the quadcopter colliding with the edge of the hoop before passing through, the overall proportion drops but only to 0.847. (Note that a collision was counted even if the quadcopter grazed the edge of the hoop as it passed through.) In other words, subjects cleanly passed through the large majority of hoops. This is not surprising due to the cost (in terms of time and loss of control) of colliding with the edge of a hoop. We added a few sentences to the manuscript to address this point (Lines 442-446).

In summary whilst the experimental design appears to have the potential for revealing some interesting findings, in it's current form this manuscript does not provide sufficient evidence/justification to support the claims made.

We thank Reviewer #2 for his many helpful comments and hope that the changes to the manuscript address his concerns.
