# Supplementary material for: Coordination of gaze and action during high-speed steering and obstacle avoidance
Source: PLoS One. 2024 Mar 8;19(3):e0289855. doi: 10.1371/journal.pone.0289855 (PMC10923441; doi:10.1371/journal.pone.0289855)
Supplement: Attachment — Submitted filename: Response To Reviewers.docx [file pone.0289855.s003.docx]

**Response to reviewers**

Manuscript number:	PONE-D-23-23553R2

Manuscript title:	Coordination of gaze and action during high-speed steering and obstacle avoidance

Authors:	Powell, Marshall, Diaz, & Fajen

We thank the reviewers for their careful reading of the revised manuscript. Our response to each comment can be found below in blue font. Changes to the manuscript are also indicated in the marked-up version. Note that line numbers below refer to those in the marked-up version of the manuscript with changes tracked. The line numbers in the unmarked version differ because MS Word includes deleted text in its count of line numbers.

**Response to Editor instructions**

Please review your reference list to ensure that it is complete and correct. If you have cited papers that have been retracted, please include the rationale for doing so in the manuscript text or remove these references and replace them with relevant current references. Any changes to the reference list should be mentioned in the rebuttal letter that accompanies your revised manuscript. If you need to cite a retracted article, indicate the article’s retracted status in the References list and also include a citation and full reference for the retraction notice.

We updated Reference 26, which was previously in press and is now published.  We also added Reference 27 to support a point that was added in response to Reviewer 3’s second comment below.

**Response to Reviewer #1 comments:**

The latest changes in the paper show a more careful consideration of limitations, a more transparent statistical analysis, and a discussion of "corner cutting behavior." The revised Anova is better explained, addressing assumptions and factors appropriately.

I'm not sure if Supporting Information is allowed, but if possible, consider presenting the additional analysis without Block 5 there. If there is no Supporting Information, maybe you find an elegant way of addressing it in the paper. If not, the current version is okay.

We added a paragraph toward the end of the section on analyses of basic performance measures (see Lines 395-401) that summarizes the analyses without Block 5.

However, I'm confused about why information about mixed linear models was removed from the end of the Data Analyses section. The paper didn't claim that Anovas were run with LME4, but MLMs (still used in the "Did subjects make anticipatory steering adjustments?" section) were mentioned.

The reviewer is correct.  The analyses in the section on anticipatory steering adjustments were run using the lmer function from the LME4 package.  We added a sentence to the Data Analysis section to tell readers which R function was used (see Lines 325-327).

The main issue that still stands out is the small sample size. It's good that the focus on a small group of skilled individuals is clearly stated in the abstract, making it easy for any reader to understand.  Overall, I appreciate the improvements in this version, especially in addressing limitations and making the statistical analysis clearer.

We are happy to hear that the reviewer is satisfied with the improvements and thank them for their helpful suggestions.

**Response to Reviewer #3 comments:**

1.The abstract of this article needs to be summarized.

We are unsure what the reviewer means by this comment.  The abstract is a summary of the article, so it is unclear to us what it would mean to summarize the abstract.  We wondered whether the reviewer meant to write “shortened” rather than “summarized” but the abstract is already under the 300-word limit, so that wouldn’t make sense.  We also wondered if they meant that the main take-away of the article needs to be summarized in the main text, but that wouldn’t make sense either since that is exactly the point of the last paragraph of the article.  We apologize for not being able to respond to this comment, but we simply don’t understand what the reviewer is asking us to do.

2.In conclusion，It is recommended that the authors point out the main future application scenarios of the main results of this paper.

We explained how the findings could inform the development of models of driver performance under conditions in which visibility is degraded, which is an important but neglected area of driver behavior modeling (see Lines 679-683).
